# Metatranscriptomic Analysis of Bacterial Communities on Laundered Textiles: A Pilot Case Study

**DOI:** 10.3390/microorganisms9081591

**Published:** 2021-07-26

**Authors:** Susanne Jacksch, Christoph König, Dominik Kaiser, Mirko Weide, Stefan Ratering, Sylvia Schnell, Markus Egert

**Affiliations:** 1 Faculty of Medical and Life Sciences, Institute of Precision Medicine, Microbiology and Hygiene Group, Furtwangen University, 78054 Villingen-Schwenningen, Germany; Susanne.Jacksch@hs-furtwangen.de (S.J.); koenig.christoph93@gmail.com (C.K.); dominik.kaiser87@gmail.com (D.K.); 2 International Research & Development–Laundry & Home Care, Henkel AG & Co. KGaA, 40191 Düsseldorf, Germany; mirko.weide@henkel.com; 3 Research Centre for BioSystems, Land Use, and Nutrition (IFZ), Institute of Applied Microbiology, Justus-Liebig-University Giessen, 35392 Giessen, Germany; stefan.ratering@umwelt.uni-giessen.de (S.R.); sylvia.schnell@umwelt.uni-giessen.de (S.S.)

**Keywords:** RNA sequencing, metatranscriptome, laundry hygiene, cotton, polyester

## Abstract

Microbially contaminated washing machines and mild laundering conditions facilitate the survival and growth of microorganisms on laundry, promoting undesired side effects such as malodor formation. Clearly, a deeper understanding of the functionality and hygienic relevance of the laundry microbiota necessitates the analysis of the microbial gene expression on textiles after washing, which—to the best of our knowledge—has not been performed before. In this pilot case study, we used single-end RNA sequencing to generate de novo transcriptomes of the bacterial communities remaining on polyester and cotton fabrics washed in a domestic washing machine in mild conditions and subsequently incubated under moist conditions for 72 h. Two common de novo transcriptome assemblers were used. The final assemblies included 22,321 Trinity isoforms and 12,600 Spades isoforms. A large part of these isoforms could be assigned to the SwissProt database, and was further categorized into “molecular function”, “biological process” and “cellular component” using Gene Ontology (GO) terms. In addition, differential gene expression was used to show the difference in the pairwise comparison of the two tissue types. When comparing the assemblies generated with the two assemblers, the annotation results were relatively similar. However, there were clear differences between the de novo assemblies regarding differential gene expression.

## 1. Introduction

A multitude of microorganisms live in modern washing machines. The common routes of contamination are worn clothing, tap water and air [1,2]. Promoted by the warm, humid and nutrient-rich environment, microorganisms such as bacteria and fungi can settle and multiply inside the machine [2,3,4]. The negative effects of such a contamination are unattractive staining, malodor and biofilm formation [2,4]. In particular, the formation of resistant biofilms might pose a risk for susceptible persons, as biofilms might represent a reservoir for (potentially) pathogenic microorganisms that re-contaminate the laundry during washing [5,6].

The microbial contamination of washing machines and laundry is further promoted by largely sustainability-driven adaptations to the washing process that are common today, such as reduced water consumption, low washing temperatures and the increased use of bleach-free liquid detergents [4].

Using the molecular approach of 16S rRNA gene pyrosequencing, we recently showed that the relatively most abundant sequence types in domestic washing machines were closely related to potentially pathogenic bacteria, such as *Brevundimonas vesicularis* or *Pseudomonas aeruginosa* inside the detergent drawer, and *Moraxella osloensis* or *Acinetobacter parvus* inside the door seal [7]. While this and other structural studies have looked at the microbial community composition of washing machines and laundry items [3,8,9,10,11,12,13,14], studies on the metabolic activities of the laundry microbiota are often limited to distinct functionalities, such as the formation and prevention of malodor [2,15,16]. Malodor is often associated with a lack of hygiene, and can negatively affect the life cycle of a textile [17].

In contrast to metagenomics studies, metatranscriptome studies unravel the totality of the genes that are expressed in a complex microbial community [18]. The next-generation sequencing of RNA (RNASeq) can determine the metabolic potential at the time of sampling by quantifying almost all of the transcripts from the present cells, and can thus help to obtain a profound insight into the expression profiles of an entire microbiota in a single experiment, characterizing the functionality of a microbial community [19,20].

Transcriptome studies are computationally challenging and usually require several bioinformatics tools [21,22]. The major steps in a typical metatranscriptome analysis include quality trimming and the removal of contaminating sequencing reads, the reconstruction of the individual transcripts, the annotation of these transcripts and genes, and the quantification of their expression [21,23,24,25]. In order to reconstruct the transcriptome, de novo assemblers based on de Bruijn graphs, such as Spades (rna mode) [26], Trinity [27], Velvet/Oases [28,29], or SOAPdenovo-trans [30], focus on the relationship between substrings of a fixed length k (*k*-mers). They can be used if no reference genome is available [22,31,32]. Each of these assemblers can produce useful assemblies, but when comparing different assembler software, a considerable degree of variability becomes evident [33,34].

In this pilot case study, we aimed to analyze the expression profiles of the post-wash bacteriota on two common fabric tissue types washed in a domestic washing machine, by generating de novo assembled transcriptomes using two different assemblers. To the best of our knowledge, our study represents the first study using metatranscriptomics for the field of laundry hygiene.

## 2. Materials and Methods

### 2.1. Sample Preparation

**Figure 1:**
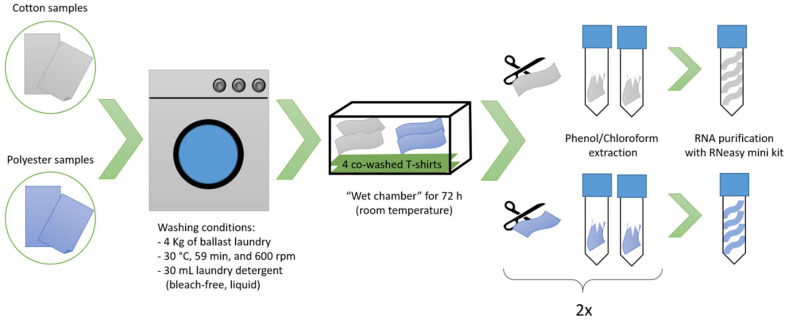
Schematic overview of a single washing experiment. Two sterile cotton (grey) and polyester (blue) textile samples (120 cm^2^ each) were washed in a standard household washing machine under mild conditions. Subsequently, the textile samples were incubated in a “wet chamber” for 72 h at room temperature. Each lobe was cut into smaller pieces under sterile conditions and distributed over two reaction tubes. After the phenol/chloroform extraction, the RNA from the two cotton and polyester lobes, respectively, were combined into a single RNA extract using the RNeasy Mini Kit. This experiment was replicated once, finally yielding two independent cotton and polyester RNA extracts each (n = 2).

Two common fabric types, cotton and polyester, were used for the washing experiments. Textile samples were cut from locally purchased, new, white, cotton and polyester T-shirts with an area of 8 × 15 cm (120 cm^2^), and were subsequently sterilized by autoclaving. In each washing experiment (Figure 1), the cut textile samples (2 × 120 cm^2^ per fabric type) were washed in a private household washing machine (an approximately five years old EcoActive W1900 appliance (Miele, Gütersloh, Germany) with approximately 4 Kg of ballast laundry consisting of worn cotton T-shirts and jean pants. A mild and short washing program for synthetics was used at 30 °C for 59 min, with a final 600 rpm spinning cycle using 30 mL of a commercial, bleach-free liquid detergent (Spee AktivGel, Henkel, Düsseldorf, Germany). The detergent was under-dosed to aid microbial survival on the washed textiles. Because many components of commercial laundry detergents are antimicrobial, we used a bleach-free liquid detergent and under-dosed it, assuming that this might increase the amount of active bacteria on the laundry after washing [35,36].

After washing, and virtually simulating “forgetting” the laundry in the washing drum, the textile samples were placed separately in a closed 38 l plastic box (Rotho Kunststoff, Würenlingen, Switzerland) and incubated at room temperature for 72 h, together with 4 pieces of washed ballast laundry (two cotton and two polyester T-shirts, with no contact with the test fabrics). This pre-incubation step was necessary to obtain sufficient RNA for the downstream analysis. Afterwards, the textile samples were stored at −80 °C until further processing.

### 2.2. RNA Extraction and Sequencing

The total RNA was isolated using a modified phenol–chloroform extraction method from Zoetendal et al. [37]. For the cell disruption, each textile sample (120 cm^2^) was cut under sterile conditions into pieces of approximately 1.5 cm^2^, and was evenly distributed into two sterile extraction tubes containing 15 sterile glass spheres (Ø 3 mm, Sigma-Aldrich, St. Louis, MO, USA) and approximately 4.3 g of a sterile ceramic silica extraction powder (Ø 0.1 mm, BioSpec Products, Bartlesville, OK, USA). Subsequently, 500 μL 10% SDS (Ambion, Carlsbad, CA, USA) and 9 mL phenol (Carl Roth, Karlsruhe, Germany) were added to each extraction tube and treated for 3 × 45 s with a FastPrep24 instrument (MP Biomedicals, Eschwege, Germany) at 5.5 m/s. Then, the extraction tubes were centrifuged for 15 min at 3220× *g* and 4 °C. After the centrifugation, the upper aqueous phase of an extraction tube was transferred to two Phase Lock Gel (PLG) Heavy Tubes (5Prime, Hilden, Germany). Then, 250 μL acid phenol and 250 μL chloroform (Sigma-Aldrich, Taufkirchen, Germany) were added to each PLG tube and gently mixed. Then, the tubes were centrifuged at 13,500× *g* for 5 min to separate the phases. The aqueous phase was transferred into new PLG tubes, and the procedure was repeated. Afterwards, the aqueous phase was transferred to a new PLG tube, mixed with 500 μL chloroform and centrifuged again at 13,500× *g* for 5 min. The supernatant was transferred to a new 2 mL tube, and the RNA was purified using an RNeasy Mini Kit (Qiagen, Venlo, The Netherlands) according to the manufacturer’s protocol. The RNA extracts belonging to the same textile sample were pooled during their application to the RNeasy column by the repeated transfer of 700 µL to the column, each followed by centrifugation. In order to exclude any contamination with DNA, a DNA digestion was performed two times for 20 min at room temperature. The final elution of the total RNA was performed with 30 μL TE buffer (Sigma-Aldrich, Taufkirchen, Germany). Two independent washing experiments were conducted (n = 2), yielding two RNA extracts from cotton and two from polyester samples, respectively.

The obtained RNA extracts were stored at −80 °C until the library preparation. For this, the RNA samples were reverse transcribed using the ScriptSeq Complete Kit for Bacteria (Epicentre, Madison, WI, USA) according to the manufacturer’s protocol. The quality of the cDNA library was determined using an Agilent 2100 Bioanalyzer (Agilent, Waldbronn, Germany). Finally, the samples were sequenced on an Illumina MiSeq platform using a MiSeq Reagent Kit v2 (both Illumina, Munich, Germany).

### 2.3. Sequence Data Analysis

The raw sequences of each single-end library were subjected to a quality control prior to their assembly. The remaining adapters and reads with a Phred quality score < 20 were removed using Trim-galore (version 0.6.6, [38]). Furthermore, human contaminations were excluded using Bowtie2 (version 2.4.2, [39]) and the human GRCh38.p13 (Release 35, [40]). In addition, ribosomal RNA was also removed from the dataset using sortmeRNA (version 4.2.0, [41]) and its provided databases. In the next step, reads with a length of less than 50 bp were discarded, truncated at a length of 280 bp and filtered out with an average Phred quality score of 20 using Trimmomatic (version 0.36, [42]). The final quality of the preprocessed reads was visualized using FastQC (version 0.11.9, [43]) and MultiQC (version 1.9 [44]). Prior to their assembly, the preprocessed reads were error-corrected using Rcorrector (version 1.0.4, [45]), as error correction is considered the best practice for transcriptome assembly [46]. In order to assemble the reads de novo to a transcriptome, Trinity software (version 2.8.5, [27]) with default parameters and Spades software (version 3.14.1, [26,47]) with the -rna-flag and different *k*-mers (13, 15, 19, 21, 25, 29, 31, 43, 55, 67, 79, 91, 103, 115, 127) were used.

The assembly statistics were calculated by the TransRate software (version 1.0.3, [48]). As mentioned in other studies, non-redundant transcripts were removed with the CD-HIT package (version 4.8.1, [49]) with an identity threshold of 95% and a word size of 10 [50,51,52]. In order to reassess the quality of the clustered transcriptome, assembly statistics were generated using Quast (version 5.0.2, [53]) and rnaQuast (version 2.0.1, [54]). In order to further determine the quality of the assembly, the read representation was calculated by aligning the input reads against the transcriptome using Bowtie2.

The transcriptome completeness was evaluated using the Benchmarking Universal Single-Copy Orthologs (BUSCO) tool (version 3.0.2, [55]) against the bacteria_odb10, archaea_odb10 (both, creation date: 2020-03-06), fungi_odb10 and eukaryota_odb10 (both, creation date: 2020-09-10) databases to quantify the percentage of single-copy orthologues. Full-length transcripts were calculated using BLAST (version 2.2.31, [56,57]) against the SwissProt/UniProtKB database (version 2020_05, [58]) with the parameters max_target_seqs 1, -evalue 1.0 × 10^−20^. The BLAST-results were analyzed using the “analyze_blastPlus_topHit_coverage.pl” script from the Trinity software. As recommended by the Trinity website, ExN50 statistics were calculated using RSEM software (version 1.3.3, [59]) with Bowtie2 and the “contig_ExN50_statistic.pl” script from the Trinity software.

In order to perform the annotation of the transcripts, open reading frames (ORFs) within the assemblies were determined using TransDecoder (version 5.5.0, [60]). The transcript ORFs with less than 150 bp were excluded from the dataset, and the BLAST (BLASTx and BLASTp; E-value: 1.0 × 10^−3^) analysis against the SwissProt/UniProtKB database and the HMMER search (version 3.3.1, [61,62]) against the PFAM database (version 33.1 (May 2020, 18259 entries [63,64]) were performed. Finally, the annotation results were loaded into the Trinotate classification tool (version 3.2.1, [65,66]) to determine the functionality by means of Gene Ontology (GO) [67]. Non-supervised Orthologous Groups (eggNOG) [68] were used to visualize the gene expression profiles grouped according to gene genealogy. Modified trinotateR [69] functions were used to evaluate the Trinotate output with R.

For the taxonomic annotation, the different assemblies were aligned against the NCBI nucleotide database (from May 2021) using BLAST (BLASTx, E-value: 1.0 × 10^−3^).

For differential expression, the transcript abundance was calculated using the “align_and_estimate_abundance.pl” and the “abundance_estimates_to_matrix.pl“ scripts from the Trinity bundle using RSEM software with Bowtie2. The differential expression analysis was carried out using R (version 3.5.3, [70]), RStudio (version 1.1.463, [71]) and edgeR (version 3.24.3, [72]). The edgeR package uses negative binomial models to detect dispersion, and later determines the differential expression with the exact test, which is analogous to the Fisher exact test [72]. An overview of the bioinformatics pipeline used here is shown in Figure S1.

## 3. Results and Discussion

### 3.1. Reads and de novo Transcriptome Assembly

In total, 8.3 million raw sequences with sequence lengths between 35 and 300 bp were obtained from the four samples after sequencing on the Illumina MiSeq instrument. After the various quality filtering steps, 6.8 million reads remained, with lengths of 50–280 bp. This corresponds to a loss of ~ 18%. A detailed listing of the numbers of sequences after the different quality filtering steps is given in Table S1.

In order to generate a de novo assembly, two different assemblers were used, i.e., Trinity and Spades. Both assemblers showed constant and good assemblies in a comparison of multiple assemblers with datasets of several different species, and were therefore selected [73]. The de novo assembly from the pre-processed reads generated 24,386 isoforms with lengths ranging from 201 to 64,155 bp using Trinity, and 13,147 isoforms of lengths between 365 and 112,899 bp using Spades.

The determined N50 value was 2192 bp for the Trinity assembly, and 2641 bp for the Spades assembly. The N50 value quantifies the average length of a contig, which comprises 50% of the sequence within the total assembly [23].

### 3.2. Evaluation of the Different de novo Transcriptome Assemblies

In order to investigate the quality of the assemblies, different measures were carried out (Table S2). First, the individual isoforms were assembled into clusters with 95% similarity, reducing the number of non-redundant isoforms to 22,321 for Trinity and 12,600 for Spades, respectively. For further quality control, the pre-processed input reads were aligned against the different clustered transcriptome assemblies to determine the read representation. In general, 80% of the reads mapping back to the transcriptome is considered an indication of a good assembly [74]. Our four sample reads aligned approximately 80–89% with each of the transcriptome assemblies.

In order to obtain an impression of the completeness of the generated assemblies, we applied BUSCO as a reference-based pipeline. This pipeline indicated that a large proportion of the single-copy genes were found in the bacterial domain. More precisely, of the 124 BUSCO groups examined, 69% were recovered from the Trinity assembly and 66% were recovered from the Spades assembly. Nonetheless, a large number of BUSCOs were missing or were too fragmented to be considered, more precisely 29% from the Trinity and 32% Spades assemblies. Although not complete, the values for the bacterial database showed good coverage of the transcripts to the known single copy orthologues, indicating an almost complete expected gene content [75]. The other sets of BUSCOs (archaea, eukaryotea, and fungi) revealed a completeness of less than 10%.

As suggested on the Trinity website, the number of full-length transcripts and the ExN50 values were determined for the further evaluation of the assembly. In order to determine the number of full-length transcripts, a BLAST analysis was first performed against the SwissProt database. To do so, a relatively low e-value (1.0 × 10^−20^) was used to store only the single best matching proteins and to discard hits for very short sequences, as these usually do not deliver a BLAST hit [76,77]. Under these conditions, 32% of the Trinity generated isoforms and 42% of the Spades generated isoforms could be assigned to SwissProt proteins, respectively. Nevertheless, out of these, 42% (Trinity) and 51% (Spades) of the near-full-length transcripts (> 70%) could be recovered with these assemblies, respectively. However, both assemblies seem to contain a high proportion of fragmented or incorrectly assembled transcripts [78].

The ExN50 value indicates the N50 value by using only the most highly expressed transcripts [79]. Both assemblers peak at low percentages, indicating a tendency to detect highly expressed isoforms (Figure S2). Therefore, under the experimental conditions used here, neither assembler could adequately detect low-expressed transcripts [80]. However, increasing the sequencing depth in follow-up experiments might enhance the ExN50 value to obtain a more complete representation of the transcriptome.

All in all, the above-mentioned indices show that the assemblies are of good quality, but the method clearly needs further improvement, e.g., by increasing the sequencing depth or by using multiple *k*-mers lengths to account for variable transcript expression [81,82].

### 3.3. Transcript Annotation

The BLAST analysis showed that bacterial sequences represented the majority (approximately 99%) of all of the sequences within the two assemblies. Eukaryotic and viral sequences were minorly abundant, probably because the ScriptSeq Complete Kit for Bacteria was used for the library preparation.

Based on the transcript counts, several bacterial genera known to be typical for washing machines and laundered textiles [7,10,13] were detected, such as *Acinetobacter* (48.5%, 51.7%), *Aeromonas* (26.1%, 21.6%), *Rhizobium* (6.0%, 6.5%), *Agrobacterium* (2.9%, 2.4%), *Moraxella* (1.8%, 2.1%) and *Pseudomonas* (0.4%, 0.4%) (the brackets show the relative abundances based on the Spades and Trinity assemblies, respectively, averaged over all of the samples). However, we also detected genera which were, to the best of our knowledge, previously not reported as being typical for washing machines or laundered textiles, such as *Sphingorhabdus* (9.9%, not detected), *Anderseniella* (2.1%, 12.1%), *Epilithonimonas* (0.9%, 1.0%), *Haematobacter* (0.5%, 0.6%) and *Escherichia* (0.04%, 0.3%). Figure S3 shows the relative abundances of the 11 most relatively abundant genera for both experiments, based on Trinity and Spades assemblies, respectively.

**Figure 2:**
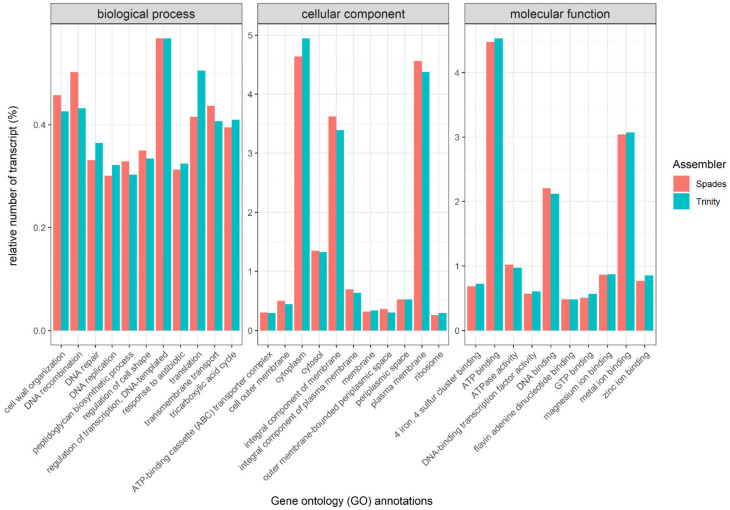
Bar chart of the assigned Gene Ontology (GO) terms of the annotated genes assembled with Spades (**red**) or Trinity (**blue**). The GO terms are categorized into biological process, cellular components, and molecular functions, respectively. For each category, the 10 GO terms with the highest relative number of transcripts are displayed in alphabetical order.

The identification of the functional classes was achieved by following the GO term classification using SwissProt gene symbols. These GO terms are divided in three categories, i.e., “biological process”, “cellular component” and “molecular functions”, which describe the attributes of a gene product [67,83]. In each of the assemblies, we detected a wide range of GO terms from all three functional categories, suggesting the active and diverse microbial gene expression of the investigated textiles (Figure 2).

In total, 3881 unique GO terms were identified with the Spades assembly, whereas 4390 unique GO terms were determined with the Trinity assembly. Both assemblies shared 3686 unique GO terms. The most frequently assigned GO terms allocated to “cellular components” were related to cytoplasm (Spades: 4.6%, Trinity: 4.9%), plasma membranes (Spades: 4.6%, Trinity: 4.4%), and the integral components of the membrane (Spades: 3.6%, Trinity: 3.4%). On the other hand, most of the “molecular functions” were associated with binding: ATP binding (Spades: 4.5%, Trinity: 4.5%), metal ion binding (Spades: 3.0%, Trinity: 3.1%) and DNA binding (Spades: 2.2%, Trinity: 2.1%). Only a small proportion of the transcripts could be assigned to “biological processes”. Here, the regulation of transcription (DNA-templated) (Spades: 0.6%, Trinity: 0.6%), DNA recombination (Spades: 0.5%, Trinity: 0.4%), and cell wall organization (Spades: 0.5%, Trinity: 0.4%) predominated. In summary, these data suggest that a metabolically active bacterial community was present on the investigated fabric patches.

Notably, despite using different assembly software, there was a high level of similarity between the assigned GO terms, which suggests the accuracy of the different assemblies and the assigned annotations [76]. Furthermore, previous research has shown that GO terms have high consistency across multiple species despite the intrinsic differences between the different assembly strategies and annotation pipelines, suggesting the usage of GO for comparisons with other studies [77].

### 3.4. Differential Expression

**Figure 3:**
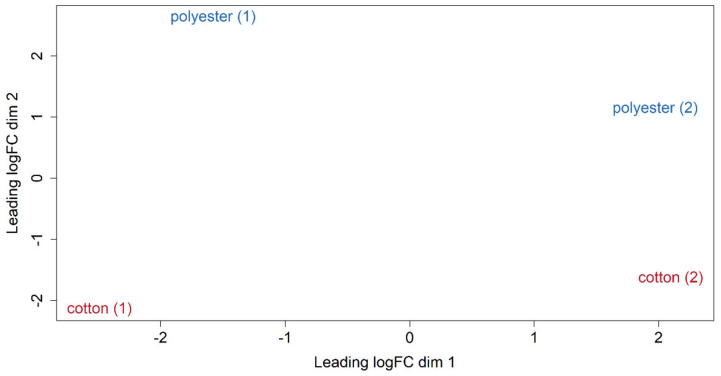
Multi-dimensional scaling (MDS) plot based on RNAseq expression profiles from different tissue type samples. The distances between the samples in the plot were calculated based on leading log2 fold changes (logFC) between each cotton and polyester sample, which were defined as the average log2-fold change for the 500 most differential genes between each pair of profiles [84]. The numbers in parentheses define the respective washing experiments. Here, the MDS plot for the Trinity assembly is shown. The very similar MDS plot for the Spades assembly can be found in Figure S4.

In order to investigate whether the tissue type had an influence on the microbial gene expression, a differential expression analysis was performed using the edgeR-R-package. After filtering and normalization using the built-in-functions of edgeR, both assemblies clearly showed a separation between the fabric types, indicating differences in their expression profiles (Figure 3). In addition, Figure 3 shows marked differences between the replicate washing experiments, strongly requiring follow-up-studies with larger sample sizes and more standardized conditions. The differences between the two experiments might originate from the different ballast laundry used. While the vertical separation of the samples seems to be textile-dependent, the horizontal separation seems to experiment-dependent.

In contrast to the annotation, the differential expression analysis showed differences between the two assemblers. Overviews of the different gene expression profiles from the different assemblers, categorized into higher functional groups, are shown in Figures S5 and S6.

Performing the differential expression, we identified 8146 genes with a log2 fold change (4447 upregulated and 3699 downregulated) for the Trinity assembly and 4037 (2248 upregulated and 1789 downregulated) for the Spades assembly when comparing the cotton and polyester samples. Out of these, 16 genes were statistically significantly differentially expressed (adjusted *p*-value < 0.01) in the case of the Trinity assembly (Table 1). Of these, nine genes were identified as being up-regulated in the cotton samples, and seven genes were identified as being down-regulated in the cotton samples. For the Spades assembly, we identified 4037 differentially expressed genes (2248 upregulated and 1789 downregulated). However, for only one gene, the expression was significantly different. It was up-regulated in the cotton samples (Table 1).

Figures S5 and S6 suggest that the number of genes with significant differences in expression between the cotton and polyester might be higher than the 17 genes detected here. In order to prove this, more standardized studies with a bigger sample size are needed. Interestingly, two of the differentially expressed genes were affiliated with *Moraxella* (Table 1), a genus which is well known for laundry malodor production [15,85].

The genes, which were significantly up- or down-regulated, were mainly enzymes that are predominantly involved in metabolic pathways, as well as the transport of substances across the cell membrane (Table 1). The identified genes are common genes that are found in different microorganisms and have a variety of cellular activities, such as the “AAA (ATPases Associated with diverse cellular Activities) family ATPases” or the “LysR family transcriptional transporter”, which is involved in virulence, metabolism, quorum sensing and motility [86,87]. In addition, our data slightly suggest that carbohydrates might have acted as substrates on the fabric samples, and might have led to differences in gene expression, as indicated by the different expression of the genes for “Sucrose-6-phosphate hydrolase” and “PTS system sucrose-specific EIIBC component”. The phosphoenolpyruvate-dependent phosphotransferase system (PTS) is found in various microorganisms, such as *Escherichia coli*, *Streptococcus mutans* and *Bacillus subtilis*, and—in addition to the transport and phosphorylation of carbohydrates—it is also involved in the movement towards carbon sources [88,89]. It is tempting to speculate that the chemical differences between cotton (made of cellulose fibers) and polyester (made of polyethylene terephthalate fibers) are responsible for these differences. Unlike natural fibers, synthetic fibers are less susceptible to bacterial degradation [90].

The observed differences in the number of differentially expressed genes may also be due to the different ways in which the used assemblers work. As there are a number of different tools and parameters that can be used to reconstruct transcripts, it is difficult to determine a single robust method [91,92,93,94]. For future studies, merging the different assemblies created with different programs and parameters might lead to a more reliable representation of the post-wash laundry transcriptome [91].

Clearly, in this pilot case study, we only worked with small sample sizes (n = 2) for each fabric type and poorly standardized washing conditions (different ballast laundry). Small sample sizes increase the variances in gene expression, resulting in lower confidence and increasing *p*-values [95]. Therefore, the number of biological replicates should be increased in future experiments, and even technical replicates should be taken into account to reduce the technical noise [78,95].

## 4. Conclusions

Our study delivered the first laundry metatranscriptome, and it suggests a differential gene expression of the post-wash bacteriota on two commonly used types of fabrics. Our data provide an initial overview and characterization of the bacterial laundry transcriptome, as well as a comparison between the two de novo transcriptome assemblers used, i.e., Trinity and Spades. Clearly, the approach needs further optimization, such as a higher sequencing depth and further biological and technical replicates, ideally in combination with DNA shotgun sequencing, in order to identify the microorganisms of which the metabolic activity shapes the microbial community on laundry. Nevertheless, the assemblies created here represent a solid basis for further metatranscriptomic studies.

## Figures and Tables

**Table 1:** Overview of the significantly differentially expressed genes. Shown here are the log fold changes (logFC), log counts per million (logCPM) and the *p*-value after the false discovery rate (FDR) correction, as calculated by edgeR for the pairwise comparison between cotton and polyester. If available, the SwissProt entry or BLAST accession number (March, 2021), as well as the organism determined by means of BLAST against the nucleotide database (May, 2021), are shown, as well as the determined up- or down-regulation of the respective gene for the cotton samples in comparison to the polyester samples. The functional orthologous groups were obtained using the EggNOG database [68]. If a protein has multiple domains, more than one functional orthologous groups is possible.

Assembly	Gene	Uniprot Entry/ Blast Accession #	Percent Identity	E-Value	Ontology	Name	Genus	log FC	log CPM	FDR	Regulation
**Trinity**	TRINITY_DN12211_c0_g1	WP_082183191.1	100.00	2.0 × 10^−08^	Energy production and conversion	FAD-binding oxidoreductase	*Rhizobium*	−9.871	5.431	0.003	down
	TRINITY_DN12794_c0_g1	WP_126090172.1	73.08	2.5 × 10^−02^	Transcription	LysR family transcriptional regulator	none available	−9.988	5.545	0.003	down
	TRINITY_DN16017_c0_g1	KIV68812.1	100.00	2.0 × 10^−36^	Carbohydrate transport and metabolism	Sucrose−6-phosphate hydrolase	*Rhizobium*	−9.852	5.412	0.003	down
	TRINITY_DN16123_c0_g1	IHFA_CHRVO	41.54	1.3 × 10^−10^	Transcription	Integration host factor subunit alpha	none available	−9.635	5.202	0.008	down
	TRINITY_DN19317_c0_g1	GLO22_ECOLI	51.90	4.3 × 10^−15^	Inorganic ion transport and metabolism	Hydroxyacylglutathione hydrolase GloC	*Rhizobium*	−9.756	5.319	0.010	down
	TRINITY_DN6555_c0_g1	WP_164056586.1	100.00	2.0 × 10^−17^	Replication, recombination and repair	AAA family ATPase	*Rhizobium*	−9.705	5.270	0.005	down
	TRINITY_DN8425_c0_g1	WP_142779495.1	100.00	1.0 × 10^−18^	Cell cycle control, cell division, chromosome partitioning	ParA family protein	*Rhizobium*	−9.602	5.170	0.008	down
	TRINITY_DN12673_c0_g1	WP_042878669.1	97.53	2.0 × 10^−25^	Amino acid transport and metabolism, Carbohydrate transport and metabolism	DMT family transporter	*Aeromonas*	9.611	5.184	0.008	up
	TRINITY_DN14275_c0_g1	WP_174060752.1	100.00	5.0 × 10^−23^	Amino acid transport and metabolism, Inorganic ion transport and metabolism	ABC transporter permease	*Rhizobium*	9.590	5.163	0.010	up
	TRINITY_DN14310_c0_g1	WP_124801776.1	100.00	6.0 × 10^−11^	Inorganic ion transport and metabolism	cation:proton antiporter	*Epilithonimonas*	9.577	5.150	0.008	up
	TRINITY_DN16876_c0_g1	YDDG_ECOLI	66.67	6.9 × 10^−08^	Amino acid transport and metabolism, Carbohydrate transport and metabolism	Aromatic amino acid exporter YddG	*Acinetobacter*	9.751	5.318	0.003	up
	TRINITY_DN16965_c0_g1	STY97430.1	95.16	1.0 × 10^−33^	Coenzyme transport and metabolism	Dihydroneopterin aldolase	*Moraxella*	9.920	5.481	0.003	up
	TRINITY_DN19448_c0_g1	WP_204155761.1	82.05	4.0 × 10^−15^	Cell motility, Intracellular trafficking, secretion, and vesicular transport	prepilin-type N-terminal cleavage/methylation domain-containing protein	*Moraxella*	9.832	5.396	0.003	up
	TRINITY_DN19763_c0_g1	Y2604_PSEAE	69.09	6.5 × 10^−21^	Cell wall/membrane/envelope biogenesis	Uncharacterized protein PA2604	*Pseudomonas*	9.985	5.544	0.004	up
	TRINITY_DN24104_c0_g1	PTSBC_SALTM	79.22	7.3 × 10^−33^	Carbohydrate transport and metabolism	PTS system sucrose-specific EIIBC component	*Aeromonas*	10.140	5.695	0.005	up
	TRINITY_DN9443_c0_g1	WP_074855682.1	100.00	4.0 × 10^−18^	Inorganic ion transport and metabolism	ArsC family reductase	*Pseudomonas*	9.803	5.368	0.003	up
**Spades**	NODE_11013_length_508_cov_2.086614_g10506	YOXD_BACSU	46.39	1.7 × 10^−35^	Function unknown	Uncharacterized oxidoreductase YoxD	*Epilithonimonas*	9.511	5.043	0.000	up

## Data Availability

All of the sequence data presented and discussed here were deposited at European Nucleotide Archive (ENA), and are available under the accession number PRJEB45608.

## Supplementary Materials

The following are available online at https://www.mdpi.com/article/10.3390/microorganisms9081591/s1. Table S1: Summary of the individual quality filtering steps using individual program specifics, as well as the number of input and output reads during each step of quality filtering. Table S2: Summary of the individual assembly statistics of the generated de novo transcriptome assemblies after clustering with CD-Hit-EST. Figure S1: Schematic overview of the de novo transcriptome reconstruction workflow and analysis pipeline procedure. Figure S2: Estimated ExN50 values of assemblies using A) Trinity and B) Spades. Figure S3: Taxonomic annotation of the different samples for the two assemblers used. Figure S4: Multi-dimensional scaling plot based on RNAseq expression profiles from different tissue types samples generated with the Spades assembly. Figure S5: Mean expression versus the log2 fold change plots (MA-plots) of the Spades assembly. Figure S6: Mean expression versus the log2 fold change plots (MA-plots) of the Trinity assembly.
