# Supplementary material for: Metatranscriptomic Analysis of Bacterial Communities on Laundered Textiles: A Pilot Case Study
Source: Microorganisms. 2021 Jul 26;9(8):1591. doi: 10.3390/microorganisms9081591 (PMC8400938; doi:10.3390/microorganisms9081591)
Supplement: Supplementary file 1 [file microorganisms-09-01591-s001.zip › microorganisms-1290707-supplementary.pdf]

microorganisms

Supplementary Materials

Metatranscriptomic Analysis of Bacterial Communities on Laundered Textiles: A Pilot Case Study

Content:

Figure/Table	Page
Figure S1	2
Table S1	3
Table S2	4
Figure S2	5
Figure S3	6
Figure S4	7
Figure S5	8
Figure S6	9

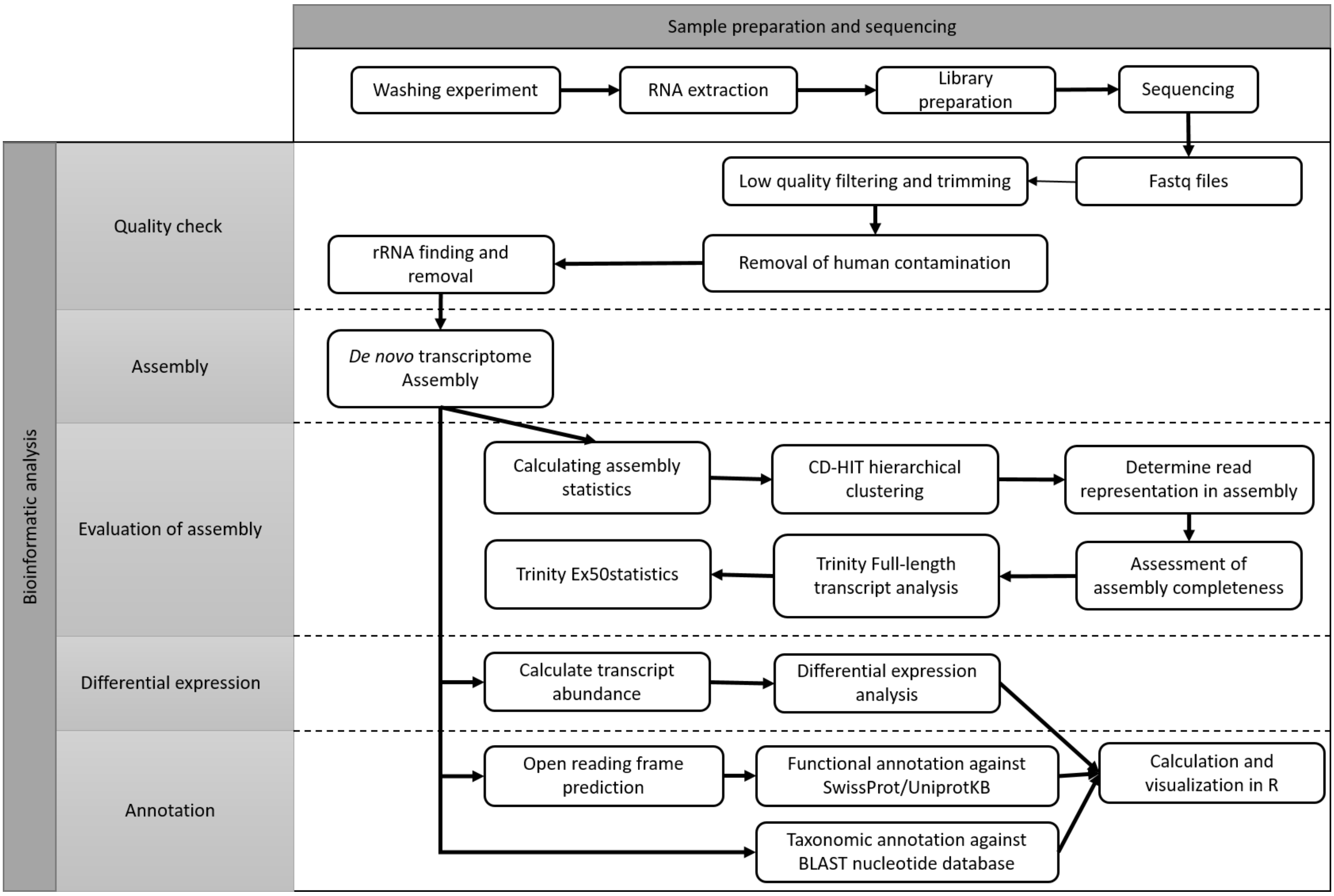

Figure S1. Schematic overview of the de novo transcriptome reconstruction workflow and analysis pipeline procedure.

Table S1. Summary of the individual quality filtering steps using individual program specifics as well as the number of input and output reads during each step of quality filtering.

Quality filtering step	Program characteristics	Cotton sample 1	Cotton sample 2	Polyester sample 1	Polyester sample 2
Raw Input Reads		2272571	2072513	1942656	2004058
Trim-galore	Input Reads	2272571	2072513	1942656	2004058
	Dropped	212044	545963	35872	130261
	Surviving reads	2060527	1526550	1906784	1873797
Human contamination (removal with Bowtie2)	Input Reads	2060527	1526550	1906784	1873797
	Aligned 0 times	96.8%	98.42%	97.97%	90.15%
	Aligned exactly 1 time	0.88%	0.39%	0.55%	4.92%
	Aligned >1 times	2.32%	1.19%	1.48%	4.93%
	Overall alignment rate	3.20%	1.58%	2.03%	9.85%
	Surviving Reads	1994595	1502357	1868081	1689357
Ribosomal RNA (removal with sortmeRNA)	Input Reads	1994595	1502357	1868081	1689357
	Rfam-5.8s-database-id98	0.00%	0.00%	0.00%	0.00%
	Rfam-5s-database-id98	0.00%	0.01%	0.01%	0.01%
	Silva-arc-16s-id95	0.00%	0.00%	0.00%	0.00%
	Silva-arc-23s-id98	0.00%	0.00%	0.00%	0.00%
	Silva-bac-16s-id90	0.38%	0.41%	0.40%	0.29%
	Silva-bac-23s-id98	0.51%	0.82%	0.54%	0.46%
	Silva-euk-18s-id95	0.04%	0.02%	0.03%	0.03%
	Silva-euk-28s-id98	0.14%	0.08%	0.14%	0.11%
	Surviving Reads	1973152	1482205	1847134	1674100
Trimmomatic	Input Reads	1973152	1482205	1847134	1674100
	Dropped	55134	25128	39437	37767
	Surviving Reads	1918018	1457077	1807697	1636333
Final Surviving Reads		1918018	1457077	1807697	1636333
Total Loss		15.6%	29.7%	6.9%	18.3%

Table S2. Summary of the individual assembly statistics of the generated de novo transcriptome assemblies after clustering with CD-HIT-EST.

Program		Spades Assembler	Trinity Assembler
Transrate	N seqs	12600	22321
	Smallest lenght	367	201
	Largest lenght	112899	64155
	N bases	20014022	21766331
	Mean length	1588.41	975.15
	N50	2494	1555
	GC (%)	50	50
Bowtie2	Read representation		
	Cotton sample 1	81.27%	80.70%
	Cotton sample 2	89.57%	89.36%
	Polyester sample 1	86.11%	85.37%
	Polyester sample 2	87.78%	87.60%
BUSCO	Bacteria (bacteria_odb10 database)		
	Complete BUSCOs	66.1%	68.5%
	Complete and single-copy BUSCOs	22.6%	26.6%
	Complete and duplicated BUSCOs	43.5%	41.9%
	Fragmented BUSCOs	1.6%	2.4%
	Missing BUSCOs	32.3%	29.1%
Trinity	Full-length transcripts estimation		
	100–90% coverage	1855	1955
	90–80% coverage	476	511
	80–70% coverage	364	474
	70–60% coverage	445	535
	60–50% coverage	448	619
	50–40% coverage	569	771
	40–30% coverage	539	836
	30–20% coverage	410	732
	20–10% coverage	208	547
	10–0% coverage	19	62
	Total contigs	5333	7042

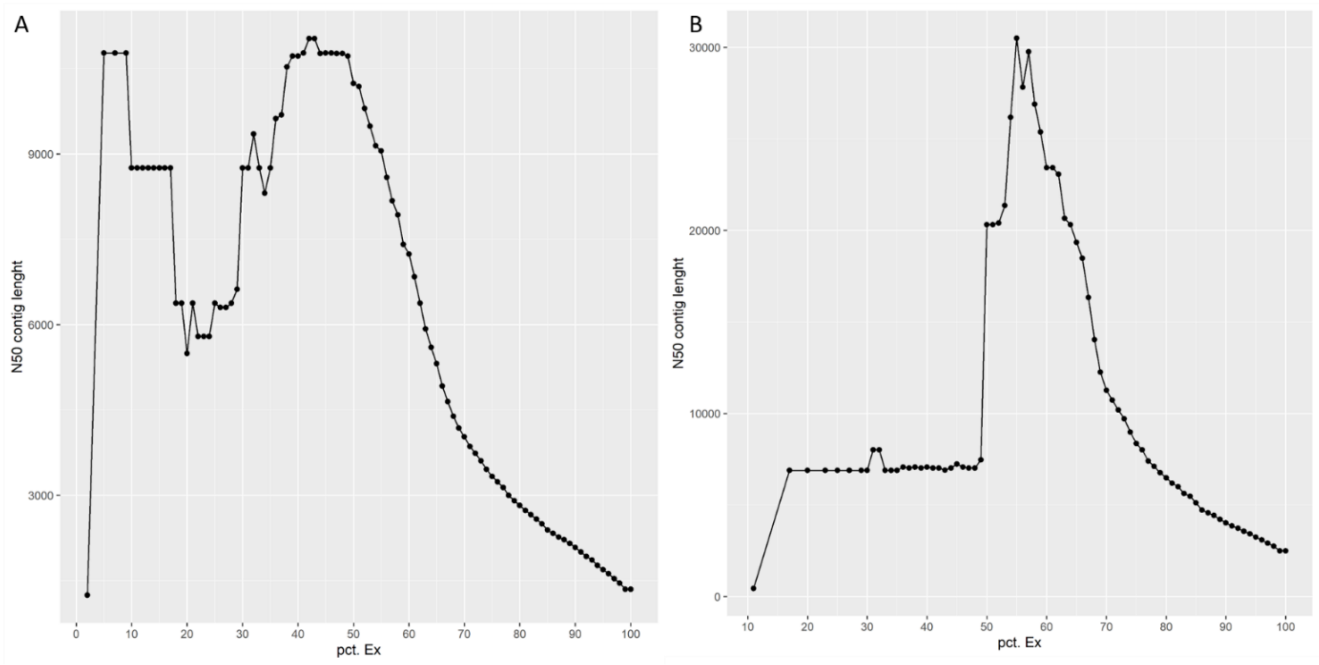

Figure S2. Estimated ExN50 values of assemblies using (**A**) Trinity and (**B**) Spades. Peaks represent ExN50 contig lengths. Peaks near 0 correspond to only highly expressed genes in the assembly and peaks near 100% correspond to low expressed transcripts in the assembly.

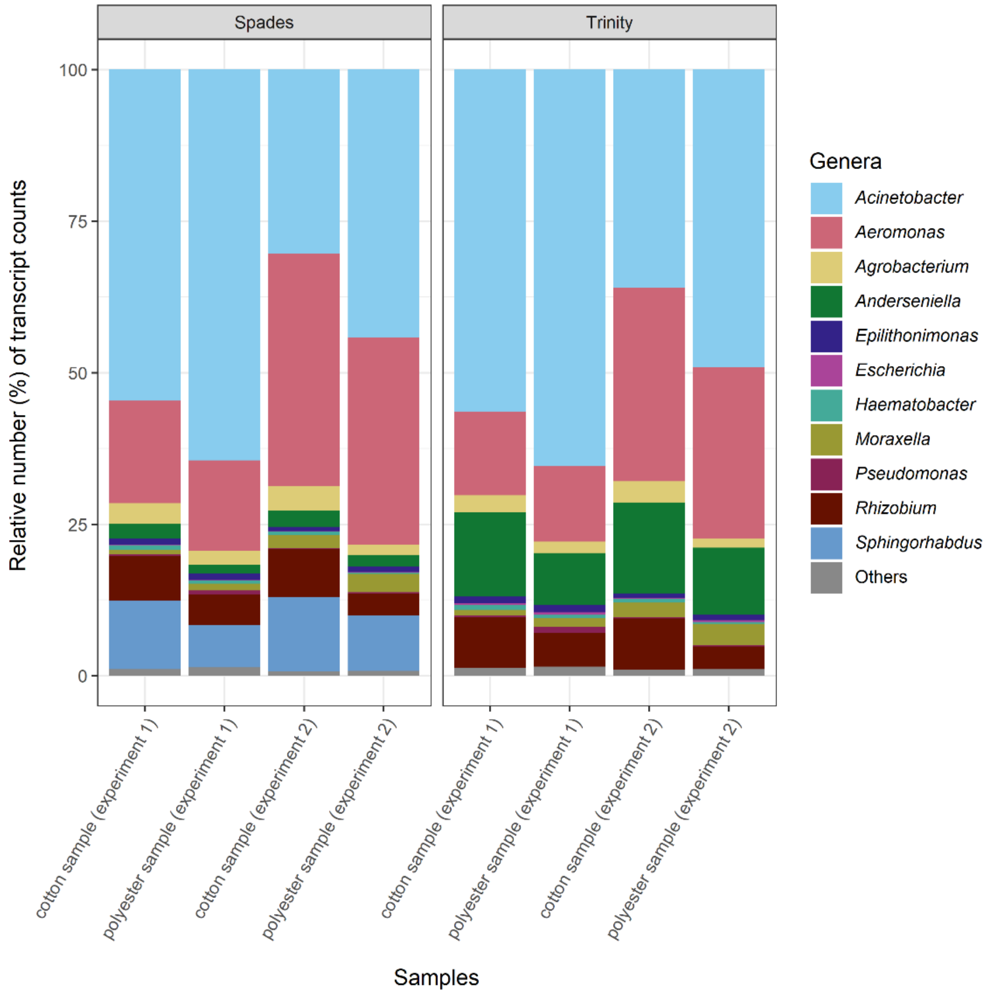

Figure S3. Taxonomic annotation of the different samples for the two assembler used. For better visualization, only the 11 genera with the highest relative number of transcripts within the respective assembly are shown, in alphabetical order.

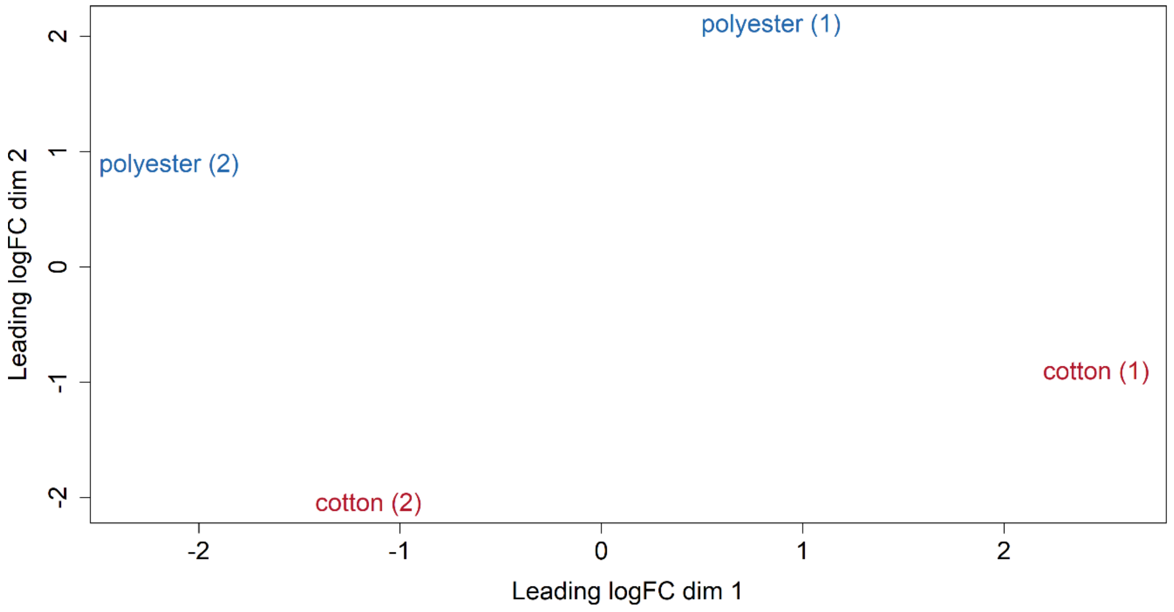

Figure S4. Multi-dimensional scaling plot based on RNAseq expression profiles from different tissue type samples generated with the Spades assembly. Distances between samples in the plot are calculated based on leading log2 fold changes (logFC) between each cotton and polyester sample. The numbers in parentheses represent the different washing experiments.

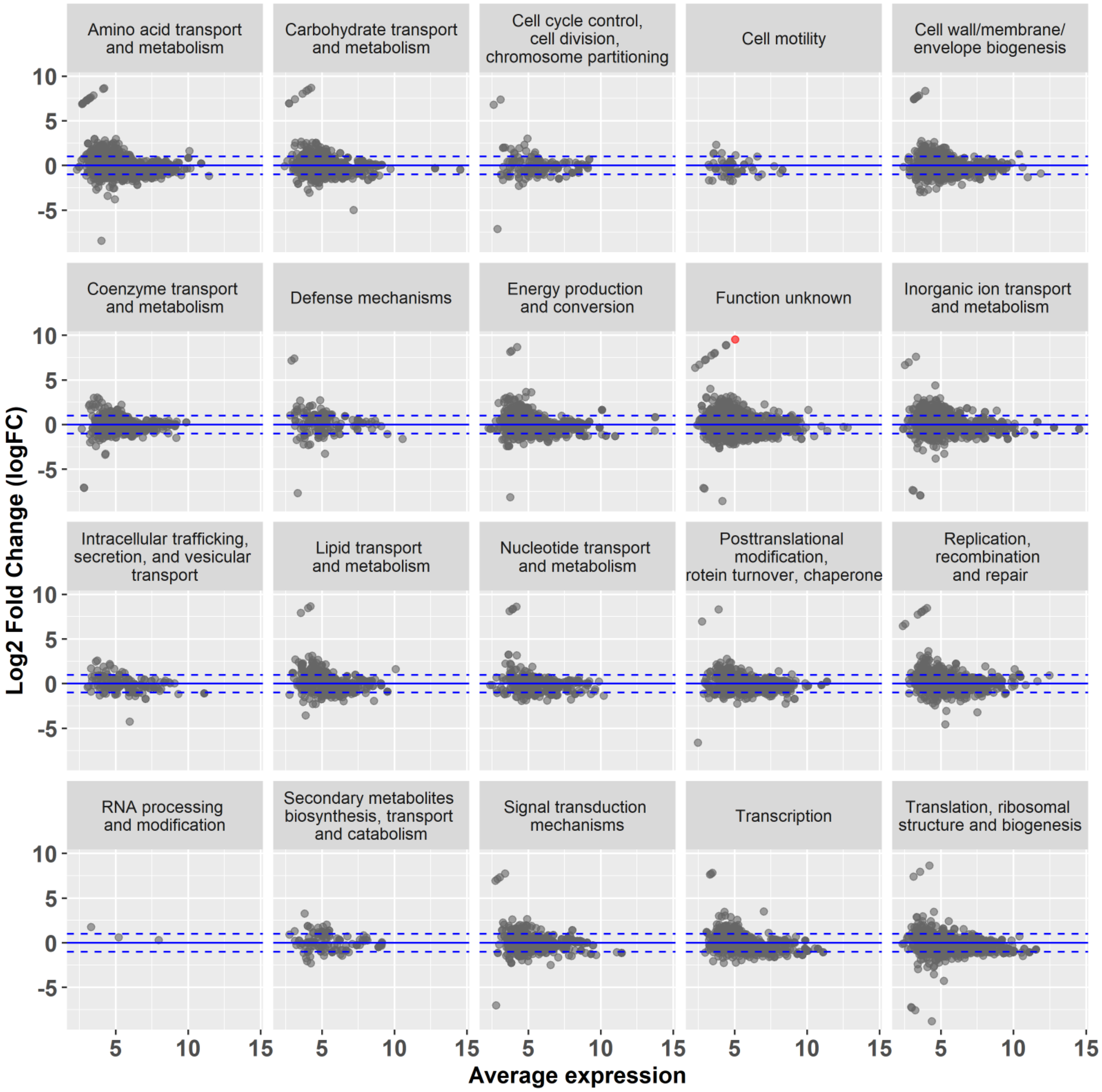

Figure S5. Mean expression versus log2 fold change plots (MA-plots) of the Spades assembly. Plot shows average expression logarithmically transformed counts per million versus the log2 fold change (logFC) for a pairwise comparison of cotton versus polyester samples. Black dots represent the individual genes. Dashed line show genes that were two-fold up or downregulated, when comparing cotton versus polyester samples. Negativ changes represent downregulated genes and positive changes represent upregulated genes. Genes that have no or only a slight change in expression are located within the area of the dashed lines. Red points show significantly differential expressed genes with adjusted p-value < 0.01.

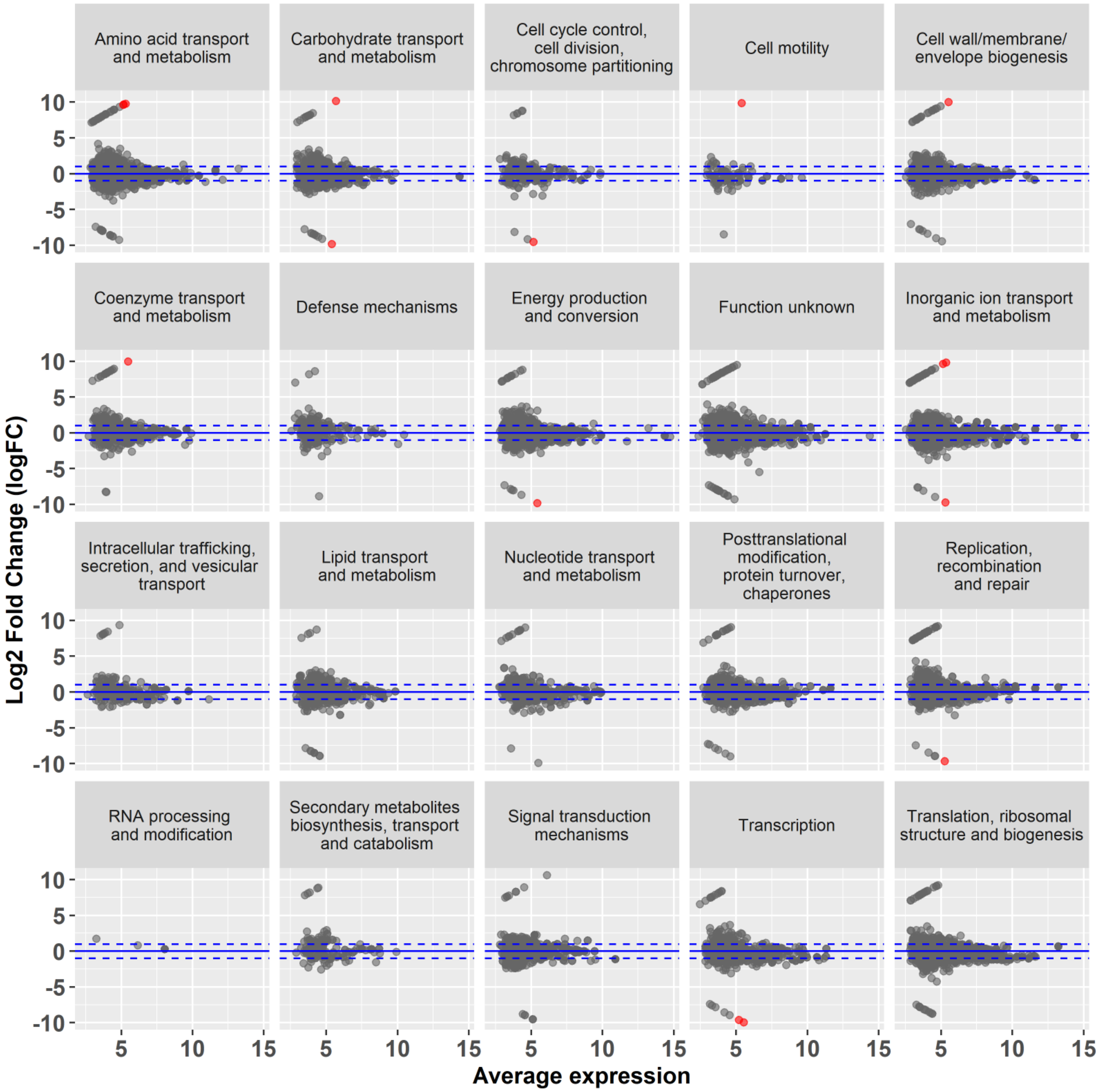

Figure S6. Mean expression versus log2 fold change plots (MA-plots) of the Trinity assembly. Plot shows average expression logarithmically transformed counts per million versus the log2 fold change (logFC) for a pairwise comparison of cotton versus polyester samples. Black dots represent the individual genes. Dashed line show genes that were two-fold up or downregulated, when comparing cotton versus polyester samples. Negativ changes representing downregulated genes and positive changes representing upregulated genes. Genes that have no or only a slight change in expression are located within the area of the dashed line. Red points show significantly differential expressed genes with adjusted *p*-value < 0.01.
